# A role for misaligned gene expression of fetal gene program in the loss of female-specific cardiovascular protection in young obese and diabetic females

**DOI:** 10.3389/fendo.2023.1108449

**Published:** 2023-02-23

**Authors:** Lakshmi Pulakat

**Affiliations:** Molecular Cardiology Research Institute, Tufts Medical Center, and Department of Medicine, Tufts University School of Medicine, Boston, MA, United States

**Keywords:** cardiac gene expression, fetal gene program (FGP), obesity, diabetes, AT2 receptor, MED13, female-specific cardiovascular protection, miR-208

## Abstract

Healthy, premenopausal women have the advantage of female-specific cardiovascular protection compared to age-matched healthy men. However, pathologies such as obesity and Type 2 diabetes mellitus (T2DM) cause losing of this female-specific cardiovascular protection in young, obese and diabetic females. Molecular mechanisms underlying this loss of female-specific cardiovascular protection in young, obese and diabetic females are not clearly elucidated. This review takes a close look at the latest advances in our understanding of sex differences in adult cardiac gene expression patterns in health and disease. Based on the emerging data, this review proposes that female biased gene expression patterns in healthy adult hearts of human and pre-clinical models support the existence of active fetal gene program in healthy, premenopausal female heart compared to age-matched healthy male heart. However, the misalignment of gene expression pattern in this female-specific active cardiac fetal gene program caused by pathologies such as obesity and T2DM may contribute to the loss of female-specific cardiovascular protection in young, obese and diabetic females.

## Introduction

The prevalence of overweight, obesity and severe obesity has increased dramatically world-wide and in the US (1–3). World Health Organization estimates that the health status of approximately 167 million people will decline by 2025 due to obesity (4). Obesity is an independent risk factor for Type 2 diabetes mellitus (T2DM) (5). Both obesity and T2DM are critical contributors to cardiovascular diseases (CVD), the number one killer in the world (6, 7). Healthy pre-menopausal women have a lower risk for CVD compared to age-matched healthy men. However, clinical evidence indicates this female-specific advantage in cardiovascular risk is attenuated by obesity and T2DM (8–11). The Framingham Heart Study shows that increase in coronary artery disease from obesity is 46% in men and 64% in women- a fact that highlights the increased risk of obese women than men for cardiovascular death (10). Young obese women have a marked increase in the hazard ratio for acute myocardial infarction (AMI), ischemic stroke (IS) and death due to cardiovascular disease with increase in body mass index (BMI) (9). A recent meta-analysis on sex differences in the association between diabetes and cardiovascular and all-cause mortality involving 5,162,654 participants shows that women with T2DM had 58% greater risk of coronary heart disease (CHD) mortality compared to men with the same condition (12). Moreover, although women are protected from atherosclerotic cardiovascular disease (ASCVD), diabetic women have similar ASCVD risk as diabetic men (13). Additionally, gestational diabetes mellitus increase cardiovascular risk of young women (14). An intriguing question is why metabolic diseases such as obesity and T2DM attenuate the female-specific protection from cardiovascular risk in young women. This mini-review summarizes the latest advances in our understanding of sex differences in cardiac gene expression and how obesity and T2DM modulate female cardiac gene expression. It also proposes that a potential misalignment of fetal gene program (FGP) induced by obesity and/or T2DM may underlie the attenuation of female-specific cardiovascular protection.

There are biological sex differences at many levels - structure, function, physiology and pathology of the heart. Echocardiography studies have shown that left ventricular (LV) mass is smaller and LV end-diastolic dimension is reduced in women than men. Compared to men, women have higher resting heart rates, longer corrected QT intervals, and higher ejection fraction at rest (15, 16). However, men respond to exercise with an increased ejection fraction than women. The inability of female heart to respond to increased demand is independent of sex differences in myocardial regulation by the sinus node or by autonomic tone (15). Preclinical studies also show that female heart is less contractile than male heart (15–17). Male and female heart differ also in fatty acid oxidation and fibrosis. Enhancing mitochondrial long-chain fatty acid uptake by Acetyl-CoA carboxylase 2 (*Acc2*) deletion in mice with pre-existing cardiac pathology is shown to improve myocardial energetics in females, but not in males (18). Conversely, cardiac fibroblasts are activated more in male hearts than in female hearts (19). Collectively these observations underscore fundamental sex differences in baseline cardiac physiology. Consistent with the sex differences in cardiac physiology, cardiac pathology also exhibits strong sex bias. Women present with a higher prevalence of myocardial infarction associated with non-obstructive coronary arteries, spontaneous coronary artery dissection, stress-induced cardiomyopathy (Takotsubo Syndrome), and heart failure with preserved ejection fraction (HFpEF) than men (15–20). On the contrary, hypertrophic and dilated cardiomyopathies occur more frequently in men than women and men are more likely to develop heart failure with systolic dysfunction and abdominal aortic aneurism than women (15–20). Sex hormones and their down-stream signaling are shown to play an important role in sex differences in cardiovascular development and disease (21, 22). However, the exact mechanisms that underlie obesity- and T2DM-induced attenuation of female-specific cardiovascular protection in young females are not yet understood.

## Sex differences in healthy human cardiac transcriptome – A bias towards genes involved in inflammation in woman’s heart

An analysis of RNA-Seq data available from the genotype tissue expression study (GTEX study) on the left ventricles (LV) collected from 46 deceased organ donors (29 males and 17 females) without any prior history of CVD has revealed some interesting sex differences in cardiac gene expression (23). A total of 178 cardiac genes were differentially expressed between men and women in the LV with up regulation of 124 genes in women and 54 genes in men respectively, indicating a significantly higher number of female-biased genes in the LV. Ingenuity pathway analysis (IPA) of these differentially expressed genes uncovered activation of signaling pathways for inflammatory response and inflammatory disease in healthy woman’s heart (23). A female bias in the expression of 30 genes related to immune system was identified. The canonical pathways showed significant female bias in activation of immune-related processes. KEGG metabolic pathway analysis also showed a female bias to the genes involved in cytokine signaling, and MGI Mammalian Phenotype indicated that mutations of these female over-expressed genes were associated with immune diseases (23). Genes overexpressed in the LVs of healthy women included genes on autosomal chromosomes encoding chemokines with inflammatory functions, specifically *CCL4*, *CX3CL1*, *TNFAIP3*, and *VCAM1* that regulates adhesion of immune cells to the endothelium. The authors concluded that the genes that were differentially expressed in healthy women’s heart were enriched in those induce inflammation (23).

## Regulation of diastolic dysfunction by sex differences in the cardiac mitochondria and the role of mitochondrial signaling in developmental programming

Diastolic dysfunction is a cardinal sign of HFpEF, a cardiac pathology that is more prevalent in women than men. Recent studies using a panel of genetically diverse inbred strains of mice (the Hybrid Mouse Diversity Panel (HMDP)), indicated that mitochondrial gene expression is highly correlated to diastolic function (24). *Acsl6* (Acyl-CoA Synthetase Long Chain Family Member 6) gene was the key determinant of diastolic dysfunction in HFpEF after integration of the data from human heart failure and studies using HMDP. The *Acsl6* expression was found to be lower in females compared to males across the HMDP (24). Men and male mice had higher expression of mitochondrial genes than females. The female hormone, estrogen, suppressed whereas the male hormone, testosterone increased mitochondrial gene expression (24). Reduced mitochondrial function in female hearts was indicated by the reduced basal and maximum respiration of cardiomyocytes isolated from female hearts compared to males. The authors concluded that the reduction in mitochondrial gene expression and function in females may contribute to the higher risk for HFpEF in women in response to high fat diet (24).

It is noteworthy that mitochondria have their own genome, that is maternally transmitted *via* highly specific mechanisms that occur during gametogenesis and embryogenesis (25). The mitochondria are responsible for more than 90% of the ATP production required for cellular functions in eukaryotic cells. Chronic intrauterine hypoxia in guinea pigs was shown to decrease mitochondrial DNA content and functional indices such as Complex (C)1-V expression and C1/CIV activity in LV tissue and cardiomyocytes of males, but not females (25). Authors proposed that chronic intrauterine hypoxia modulates intrinsic properties of specific mitochondrial respiratory complexes as a programming mechanism of cardiac dysfunction in the offspring. Thus, the female-specific mitochondrial protection observed during chronic intrauterine hypoxia may play a role in female-specific cardiac protection observed in pre-menopausal females. Literature shows that various environmental factors and changes in maternal diet and metabolic health during the preconceptional and early gestational periods modulate mitochondrial number, DNA content and function in mice and humans. These observations have lead to the proposal that mitochondria may represent a key cellular target underlying developmental programming (26).

## Potential role of fetal gene program in female-specific cardiovascular protection in healthy young female

Though human cardiac transcriptome analysis of healthy donors showed that healthy female heart has activation of inflammatory pathways (23), a close look at this gene expression pattern actually indicates the existence of active FGP in healthy woman’s heart. The gene expression pattern in fetal tissues during development is termed as FGP and this gene expression pattern changes in postnatal heart as the heart matures and handles the increased volume and work load required to maintain proper circulation throughout the growing body (27, 28). Thus, it is surprising that the autosomal genes that showed female specific increased expression in adult human hearts, *CCL4*, *CX3CL1*, *TNFAIP3* and *VCAM1*, are also required during fetal development. For example, *CCL-4* and *CX3CL1* are involved in human trophoblast migration at the fetal-maternal interface (29) and *CX3CL1*is needed for implantation of blastocyst (30). The role of *TNFAIP3* in development is highlighted by the fact that haploinsufficiency of *TNFAIP3* causes a rare autoinflammatory disease (HA20), and *TNFAIP3* is critical for the development of monocyte derived cells in lymphoid organ and of microglia in the central nervous system (31, 32). Finally, *VCAM1* is essential for chorioallantoic fusion and placentation (33). Thus, the genes that are overexpressed in the LV tissues of healthy women are part of the FGP and required for normal embryonic development.

We reported on the sex differences in some of the cardiac biomarkers in healthy male versus female Zucker lean (ZL) rats and healthy (ZL) versus obese and hyperglycemic [Zucker Diabetic Fatty (ZDF)] rats (34). At the age of 5 months, the heart tissues of healthy female ZL rats showed increased expression of *Agtr2* that codes for the Angiotensin II type 2 receptor (AT2R), an X-linked gene, and Mediator Complex Subunit 13 (*Med13*, also known as *Thrap1*), an autosomal gene located on Chromosome 1 compared to healthy male hearts (34). *Agtr2* is an anti-inflammatory and reparative gene implicated in cardiovascular protection (35–37). The highly abundant angiotensin II receptor protein expressed in rat fetus is AT2R indicating that *Agtr2* is part of fetal gene program and knocking out *Agtr2* increases high fat diet-induced kidney injury (38, 39). Moreover, loss of *Agtr2* attenuated insulin sensitivity in female mice, but not in male mice (40), and reduced the ratios of heart and kidney to the body weight in mice born to dams fed with a low protein diet, implying that AT2R signaling is involved in healthy development of offspring when the mother is subjected to nutritional stress (41). MED13 is a subunit of the cyclin-dependent kinase 8 (CDK8) kinase module in the eukaryotic mediator complex and regulates cell cycle, development and growth. Improtantly, MED13 regulates zygotic genome activation and is needed for post-implantation development of mice (42) indicating the role of MED13 in FGP. Mutations in *Med13* are implicated in neurodevelopmental disorders including developmental and epileptic encephalopathy with infantile spasms (43, 44). *Med13* is also a regulator of diet-induced obesity (45, 46). Thus, the two genes, *Agtr2* and *Med13*, that showed sexual dimorphism with a female bias in healthy rat hearts are genes involved in FGP.

MED13 mRNA is a target of microRNA miR-208a (45, 46). miR-208a is a cardiac specific miRNA that is encoded within an intron of α-cardiac muscle myosin heavy chain gene (*Myh6*). MiR-208a is a member of a miRNA family that also includes miR-208b, encoded within an intron of β-cardiac muscle myosin heavy chain gene (*Myh7*). Heart-specific transgenic overexpression of miR-208a resulted in cardiac hypertrophy and suppression of both MED13 and myostatin 2 that negatively regulate muscle growth and hypertrophy and induced arrhythmias in mice (47). Conversely, knocking out miR-208a resulted in loss of P waves preceding QRS complexes in ECG recordings indicating that loss of miR-208a caused atrial fibrillation. Consistent with impaired atrial conduction, miR-208a^-/-^ mice exhibited significantly prolonged PR intervals in ECGs compared to wild type mice (47). Therefore, while cardiac overexpression of miR-208a causes hypertrophy and pathological remodeling, baseline expression of miR-208a is needed to maintain normal atrial conduction. Moreover, studies on human fetus showed that miR-208a expression in human fetal heart positively correlates to the expression of proliferation marker Ki67 indicating its role in FGP (48). Interestingly, miR-208a expression also exhibited sexual dimorphism in healthy rat hearts with a female bias (34). Thus, miR-208a is another gene that is part of FGP and exhibit sexual dimorphism in adult rat heart.

Although at birth the average human female heart weight is 5% larger than the male heart, at adulthood, the average woman’s heart weight is 26% smaller than that of man (49, 50). Analysis of sexual dimorphism in miRNA-mRNA networks showed that the miRNAs that showed sexual dimorphism in healthy humans and mice were not the same as those showed sexual dimorphism in humans and mice with cardiovascular pathologies (51). Authors pointed out that the miRNAs that showed sexual dimorphism were not enriched on sex chromosomes and proposed that ‘a dedicated genetic program’ that creates the sexual biases of these miRNAs’ exists. Their analysis of miRNA-mRNA networks revealed that male biased network over-represented GOs such as angiogenesis whereas female-biased network over-represented GOs such as heart development (51). This observation further supports the idea that the miRNA-mRNA network with female bias in adult hearts indicate active FGP in adult female heart.

It is important to note that although female heart is smaller than that of male in multiple species, female offspring’s heart coped with the intrauterine hypoxia stress better than the male offspring as shown in the guinea pig study (25). Although studies that look at sexual dimorphism in the cardiac gene expression in healthy humans and pre-clinical models are limited, the data emerging from these studies have identified a female biased increased cardiac expression of genes in young healthy females that have critical roles in fetal development. As explained above, genes that show female biased expression in healthy heart such as *CCL4*, *CX3CL1*, *TNFAIP3*, *VCAM1*, *Agtr2*, *Med13*, and microRNAs have critical roles in fetal and/or early development. Collectively these findings strongly suggest existence of active FGP in adult female heart. This review proposes that the presence of this active fetal gene program in female heart contributes to the female-specific cardiovascular protection seen in healthy premenopausal females.

## Discussion

Recent studies show that sex chromosome dependent cardiac sex differences that are part of FGP arise even before gonad formation (52). Evidence also suggest that FGP is reactivated in cardiac pathologies caused by metabolic and biochemical assaults including hypoxia, diabetes, hypothyroidism, ischemia, hypertrophy, atrophy etc. (53–55). In this context, similarities in the expression patterns of myosin heavy and light chains, actin, Troponin and Titin during fetal cardiac development and cardiac disease have been summarized previously (54). The repression of potassium channels is also similar in fetal heart and in heart failure patients. Similarly, atrial natriuretic peptide (ANP) and brain natriuretic peptide (BNP) are expressed in ventricles during fetal cardiac development and in heart failure. Drugs that increase these fetal gene proteins such as SERCA2 (56, 57), and ANP and BNP (58) improve cardiac functions in cardiac pathologies. We reported that increasing *Agtr2* gene and AT2R protein expression by the AT2R-specific peptide ligand NP-6A4 in obese and diabetic male heart improves cardiac functions and cardiac capillary density and mitigates cardiac hypertrophy and fibrosis (35). All of these examples strongly support the notion that re-activation of cardiac fetal gene program in heart disease is an adaptive mechanism to protect the heart under stress. Thus, an intriguing question is that how the active fetal gene program in healthy young female’s heart that contributes to the female-specific protection from cardiovascular diseases is modulated in young, obese and diabetic females who lose such protection. A partial answer to this question is evident in the cardiac gene expression pattern of *Agtr2*, *Med13* and miR-208a in healthy versus obese and diabetic female rats.

### Misaligned gene expression of fetal gene program in young, obese and diabetic female heart

**Figure 1:**
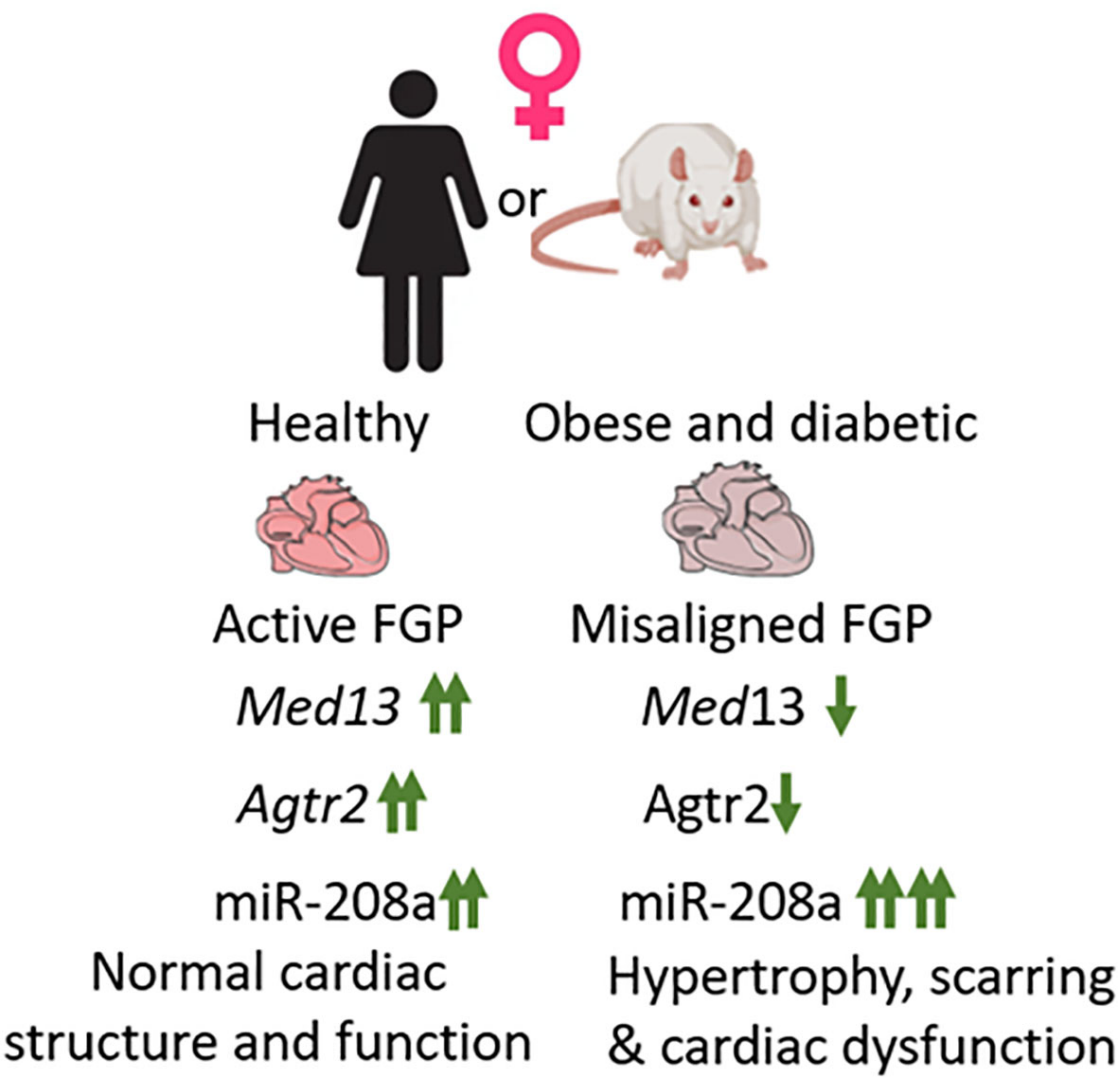
Healthy female heart exhibits active fetal gene program (FGP). Three genes that are part of fetal gene program, *Med13, Agtr2* and miR-208a exhibit increased expression in healthy female heart compared to healthy male heart (marked↑↑). In response to obesity and diabetes, in female heart both *Med13* and *Agtr2* expression becomes, suppressed and miR-208a expression increases, thus causing a misalignment of FGP. This results in increased hypertrophy and scarring in obese and diabetic female heart and increase the risk for heart failure.

Comparison of expression patterns of these genes between healthy (ZL) and obese and diabetic (ZDF) rats showed that expression of both *Agtr2* and *Med13* were significantly suppressed in female ZDF rats compared to age-matched healthy female ZL rats (34). *Med13* was also suppressed in male ZDF rats compared to healthy male ZL rats, but *Agtr2* expression remained unchanged in male ZDF rats. Conversely, miR-208a expression almost doubled in both male and female ZDF rats compared to healthy male and female ZL rats respectively. However, since the miR-208a expression was already several fold higher in healthy female ZL rats compared to male ZL rats, the additional increase in miR-208a expression in ZDF female rats resulted in about10 fold higher expression of miR-208a in female ZDF rats than that seen in male ZDF rats (34). Thus, there is a clear misalignment of cardiac expression of cardioprotective AT2R (suppressed only in ZDF female) and cardio-detrimental miR-208a (about10 fold higher expression in ZDF female compared to ZDF male) (**Figure 1**). Consistent with this misalignment in the cardiac expression of these genes (*Agtr2*, *Med1*3 and miR-208a) that are part of fetal gene program, the 5-month old female ZDF rat exhibited cardiac hypertrophy and scar tissue in the heart (34) whereas such cardiac structural damage was not observed in age-matched male ZDF rats (34).

In conclusion, this review proposes that female-specific cardiovascular protection is, in part, due to the presence of an active fetal gene program in young, healthy female heart. However, development of obesity and diabetes causes misalignment of this fetal gene expression pattern that predisposes obese and diabetic young females to lose their female-specific cardiovascular protection.

## Author contributions

The author confirms being the sole contributor of this work and has approved it for publication.
